# The mircrine mechanism controlling cardiac stem cell fate

**DOI:** 10.3389/fgene.2013.00204

**Published:** 2013-10-10

**Authors:** Toru Hosoda

**Affiliations:** Institute of Innovative Science and Technology, Tokai UniversityIsehara, Kanagawa, Japan

**Keywords:** mircrine, microRNA, c-kit, cardiac stem cells, gap junctions

## Abstract

The recent identification of c-kit-positive cardiac stem cells revealed the great growth reserve of the heart, in which connection among cells might be essential in regulating their fate. Especially, the mircrine mechanism, translocation of microRNAs (miRs) from a cell to another via gap junctions, appeared to be important in controlling the differentiation of cardiac stem cells. The modification on miR expression and/or translocation may be able to enhance further the clinical efficacy of cellular therapy.

## INTRODUCTION

**FIGURE 1:**
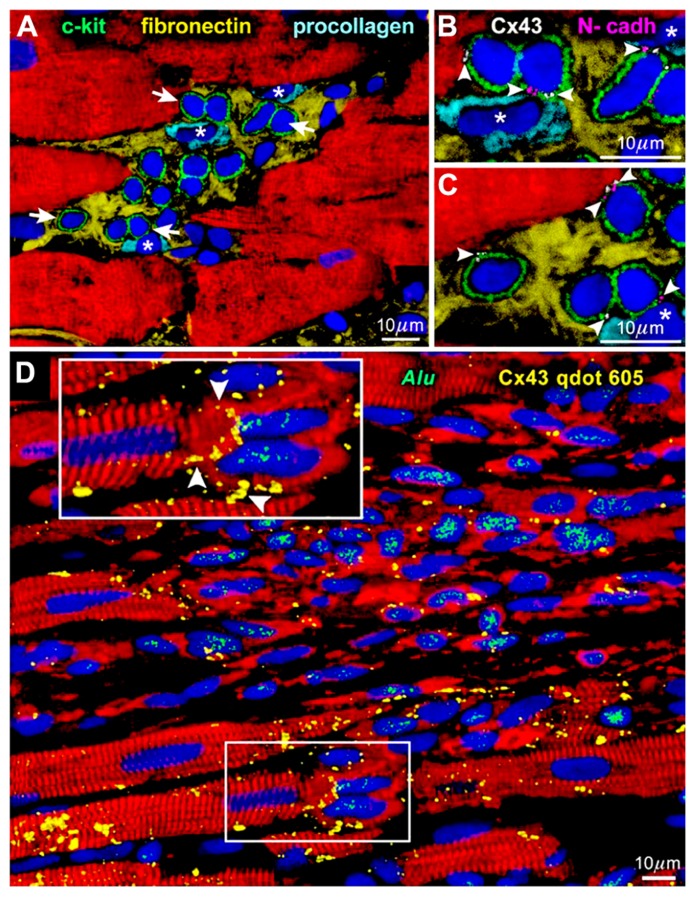
**Niches in the normal human heart (Bearzi et al., 2007).** **(A)** c-kit-positive human cardiac stem cells (green) are clustered in interstitial spaces filled with fibronectin. Arrows in **(A)** define the areas in **(B,C)**. **(B,C)** At a higher magnification, gap (connexin 43: Cx43, white) and adherens (N-cadherin: N-cadh, magenta) junctions are detected between stem cells (green) and supporting cell, i.e., myocytes (red) and fibroblasts (*). **(D)** Connexin 43 (Cx43, yellow) is present between regenerated human myocytes (red; *Alu*, green) and spared rat myocytes (red; *Alu*-negative). See inset for a higher magnification.

The heart had been considered post-mitotic and terminally differentiated. However, the identification of resident c-kit-positive cardiac stem cells in the adult mammalian heart, especially in humans (Bearzi et al., 2007), has challenged this long-lasting dogma; the myocardium turned out to be continuously and dynamically renewed by the activation, migration, and differentiation of the stem cell compartment (Kajstura et al., 2010). In general, tissue stem cells are stored in a specialized structure called “niche” and protected from external unfavorable stimuli (Fuchs et al., 2004). In the human heart, cardiac stem cells exist in the interstitial spaces among matured cardiomyocytes, namely “cardiac niches” (**Figure 1A**), where the primitive cells form gap and adherens junctions with surrounding myocytes and fibroblasts (**Figures 1B,C**) that function as supporting cells in the structure (Bearzi et al., 2007).

## MYOCARDIAL REGENERATION BY CARDIAC STEM CELLS

When c-kit-positive human cardiac stem cells are injected to the area close to the infarcted myocardium of immunosuppressed rats, muscles and vessels are regenerated improving significantly the ventricular function (Bearzi et al., 2007). In a detailed observation, gap junctions are formed between the newly formed human cardiomyocytes and pre-existing rat myocytes (Figure 1D). This connection should be responsible for the functional integrity of the regenerated tissue, in which arrhythmia is hardly inducible (Goichberg et al., 2011) in spite of the variance in size among human and rat cells.

Although the number of the regenerated cardiomyocytes may exceed that of the lost muscle cells of the host (D’Amario et al., 2011), those found in the infarcted area are usually small and resemble fetal or neonatal myocytes. Meanwhile, stem cells eventually engrafted and differentiated in the remote and surviving regions may mature in a short-term and become indistinguishable from the surrounding myocytes (Dawn et al., 2005). Together with the fact that the differentiation of stem cells gets promoted upon co-culture with parenchymal cells, it implies as if the presence of fully matured cardiomyocytes next to the committed stem cells is fundamental for the progressive maturation of the primitive population.

## RNA INTERFERENCE BY microRNAs

MicroRNAs (miRs) are a class of small non-coding RNAs that are evolutionally well-conserved and negatively regulate gene expression by repressing protein translation and/or by promoting mRNA degradation. These short RNAs are not uniformly distributed in the organism but show a preferential localization that is organ and cell specific. One miR is considered to target hundreds of mRNA, whereas one mRNA is interfered by multiple miRs; this complex network is regulating numerous biological processes including development, propagation, specification, and senescence. In the cardiovascular system, the roles of many miRs have been disclosed (Small et al., 2010), and among them, microRNA-499 (miR-499) is barely detectable in c-kit-positive cardiac stem cells while cardiac myocytes express at a high level (Hosoda et al., 2011) together with other miRs such as miR-1 and miR-133. This distinctive distribution may indicate its importance in the specialization process of cardiomyocytes.

As for the targets of miR-499, genes involved in differentiation, *SOX6* and *PTBP3* hold in their 3′-untranslated regions (3′-UTRs) well-conserved miR-499 binding sequences. In fact, whereas the transcripts of these genes are abundant in cardiac stem cells and cardiomyocytes, the corresponding protein expressions are minimal only in the myocyte compartment (Hosoda et al., 2011) suggesting the translational inhibition of *SOX6* and *PTBP3* genes by miR-499. This interaction was further confirmed by the *in vitro* reporter system; 3T3 fibroblasts, which do not possess miR-499, were transfected with plasmids overexpressing miR-499 together with reporter plasmids in which the 3′-UTRs of *SOX6* and *PTBP3* were respectively ligated downstream of the luciferase coding sequence. The luciferase activity decreased in a dose-dependent manner (Hosoda et al., 2011) suggesting that these two genes are actual targets of miR-499.

## THE MIRCRINE PHENOMENON

When rat neonatal myocytes, serving as the “donor” cells, and enhanced green fluorescent protein (EGFP)-labeled human cardiac stem cells, representing the “recipient” cells, were co-cultured for 36 h, *in situ hybridization* revealed miR-499 appearing in 35% of the recipient cells; in the presence of the gap junction inhibitor, however, the percentage decreased to less than half (Hosoda et al., 2011). At the interface of these two types of cells, connexin 43 was detected implicating potential translocation of miR-499 via gap junction channels. The amount of miR-499 in cardiac stem cells after 5 days of co-culture measured by quantitative RT-PCR was ~200-times more than that of those cultured alone (Hosoda et al., 2011). However, the exposure of stem cells to the medium obtained from myocyte cultures failed to increase miR-499 expression further documenting the necessity of the direct contact of these cells.

**FIGURE 2:**
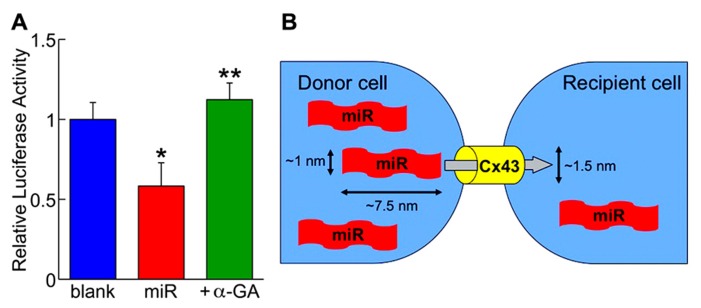
**(A)** The repressive activity of translocated miR-499 (miR) on the 3′-untranslated region of *SOX6* gene is abolished by the gap junction inhibitor (+α-GA). Blank indicates donor cells transfected with empty plasmid. *P < 0.05 vs. blank; **P < 0.05 vs. miR. **(B)** Schematic representation of the “mircrine” mechanism: the translocation of miRs from a donor cell to a recipient cell via gap junctions.

In order to visualize the translocation of miRs, fluorochrome Cy3-labeled miR-499 was synthesized and microinjected to human cardiac stem cells in culture. Under the microscopic observation, the fluorescent dye was transferred from the injected cell to the neighboring cells (Hosoda et al., 2011). More importantly, miR-499-overexpressing human cardiac stem cells considered as the “donor” was co-cultured with those expressing luciferase conjugated to the 3′-UTR of *SOX6* serving as the “recipient.” After 3 days, the luciferase activity was attenuated by 40%, which was prevented by adding the gap junction inhibitor (**Figure 2A**; Hosoda et al., 2011). These data clearly demonstrate the preserved function of the translocated miRs as well as the dependency on gap junctions. This new modality of cell-to-cell communication, different from paracrine, was termed “mircrine” (**Figure 2B**).

## miR-499 AND THE CARDIAC STEM CELL FATE

By overexpressing miR-499 in cardiac stem cells, the amount of SOX6 and PTBP3 proteins decreases. This modification is accompanied by augmented differentiation of the cells into myocyte lineage as documented by the expression of myocyte-specific antigens NKX2-5 and GATA4. Additionally, the introduction of siRNA against *SOX6* and *PTBP3* genes, respectively, enhances similarly the myocytic commitment, which indicates the function of miR-499 through *SOX6* and *PTBP3* inhibitions (Hosoda et al., 2011). When infarcted rat myocardium received infusion of c-kit-positive human cardiac stem cells overexpressing miR-499, the size of the regenerated human cardiomyocytes was larger and more matured compared to that found in the heart treated with naive cardiac stem cells. Both treated hearts showed improved cardiac function than untreated, but the one with miR-499 overexpression experienced a greater therapeutic effect (Hosoda et al., 2011).

## CLOSING REMARKS

Recently, c-kit-positive cardiac stem cells were used clinically. In this SCIPIO trial, autologous stem cells were infused into the coronary artery bypass graft (CABG) vessels of the severe heart failure patients 4 months after the operation. Compared with the control group treated with bypass surgery only, the cellular therapy resulted in the remarkable augmentation of cardiac performance accompanied by the improved symptom (Bolli et al., 2011). All 20 patients were successfully treated without jeopardizing patients’ safety (Chugh et al., 2012). In the future, by modifying miR-499 expression and/or translocation, the cardiac stem cell therapy could be advanced. Moreover, the mircrine mechanism might be operable in controlling the fate of various cells in different organs. Further investigations and progresses are expected in the field.

## Conflict of Interest Statement

The author declares that the research was conducted in the absence of any commercial or financial relationships that could be construed as a potential conflict of interest.
